# Multi-omics integration to identify immune-associated biomarkers and potential therapeutics in periodontitis

**DOI:** 10.3389/fmed.2025.1640961

**Published:** 2025-10-10

**Authors:** Ling Jin, Zhong-zheng Yuan, Yin Liu

**Affiliations:** ^1^The First Outpatient Department, Stomatological Hospital of Jilin University, Changchun, China; ^2^Wuxi Stomatological Hospital, Wuxi, Jiangsu, China

**Keywords:** periodontitis, immune microenvironment, DNA methylation, machine learning, diagnostic biomarkers

## Abstract

**Background:**

Periodontitis, a chronic inflammatory disease of periodontal tissues, is linked to immune response and epigenetic modifications, with DNA methylation playing a crucial role. This study integrates transcriptomic and DNA methylation profiles from periodontitis patients to explore the immune microenvironment and identify potential biomarkers and therapeutic targets.

**Methods:**

Transcriptomic and methylation profiles from 24 periodontitis patients were analyzed to evaluate the immune microenvironment and identify related abnormal genes. WGCNA was used to identify immune cell-associated genes. Subsequently, machine learning algorithms identified diagnostic biomarkers for periodontitis, which then validated in two cohorts with 247 and 310 periodontitis patients, respectively. Finally, network pharmacology analysis identified potential targeted drugs for the candidate genes.

**Results:**

We obtained 23,528 differentially methylated sites and 1,641 differential expressed genes. Immune cell analysis identified eight abnormal cell types in periodontitis, and WGCNA highlighted two gene modules linked to these immune alterations. Machine learning with random forest and SVM identified nine key genes (ATP2C2, FAM43B, FOXA3, HSPA12A, KIF1C, NCS1, PGM1, RASSF6, SH2B2) with diagnostic efficacy, achieving high AUC scores across validation datasets. Network pharmacology analysis identified three drugs—bisphenol A, acetaminophen, and valproic acid—as potential regulators of these genes, offering new treatment avenues.

**Conclusion:**

Through integrating s transcriptomic and DNA methylation profiles, nine genes have been filtered as potential diagnostic biomarkers of periodontitis. Drugs targeting these genes may serve as potential therapeutics for periodontitis. These findings reveal valuable insights into immune and epigenetic mechanisms in periodontitis, presenting new biomarkers and therapeutic options that may enhance clinical diagnosis and treatment of the disease and provide unique insights for further exploration of the pathogenesis of periodontitis and the development of related therapeutic drugs.

## Introduction

Periodontal disease is considered to be the most common disease in humans. The prevalence of periodontal disease is showing a significant increase (1), and globally, the prevalence of severe periodontal disease is 11%, affecting 743 million people (2). Epidemiologic surveys have shown that the leading cause of tooth loss worldwide is periodontitis, which is associated with a reduced quality of life and may cause a variety of other systemic health problems (3). Periodontitis is a chronic inflammatory condition affecting the tissues that support teeth, initiated by plaque buildup. This process results in progressive tissue destruction, formation of periodontal pockets, loss of attachment, and resorption of alveolar bone, ultimately causing tooth mobility, gum recession, and eventually, tooth loss (4). Previous studies have reported the complex molecular mechanisms of this periodontitis (5). However, the specific roles of genes, cell types, and cellular mechanisms in the development of periodontitis remain unclear, and there are currently no reliable early diagnostic markers or therapeutic targets available (6, 7). For instance, researchers found that chronic injury may alter transglutaminase gene expression, potentially playing a crucial role in remodeling and adaptation (8); It has been found that a significant link between miRNA in gingival sulcus fluid and the risk of periodontitis (9).

While bacteria are essential in initiating periodontitis, disease progression largely relies on the host’s immune response. An excessive or imbalanced immune reaction to these microorganisms can speed up both the onset and advancement of periodontitis (10), accompanied by the release of various inflammatory mediators and cytokines (11). For example, prostaglandin E_2_ (PGE_2_), interleukin-1β (IL-1β), tumor necrosis factor-*α* (TNF-α) (12), IL-8 (13), and interferon-*γ* (IFN-γ) (14). Thus, the immune response of the host, particularly the cellular immune response, is crucial in regulating the equilibrium between the repair and damage of periodontal tissues (15). Therefore, current research on periodontitis focuses on understanding how the immune system and immunomodulatory factors influence periodontal inflammation and alveolar bone degradation, as well as the role of molecular regulatory networks in immune cell activation and differentiation (4).

To further elucidate the mechanisms underlying periodontitis, it is important to consider not only the immune response but also the epigenetic factors that regulate gene expression. Epigenetics refers to changes in gene expression that do not involve alterations to the underlying DNA sequence. Key epigenetic processes include DNA methylation, histone modifications, and chromatin remodeling. Recent studies suggest that chronic inflammatory conditions, such as periodontitis, can induce epigenetic changes, thereby modulating the immune response and contributing to disease progression. Growing evidence indicates that these epigenetic changes are linked to the development of periodontitis (15). In particular, epigenetic modifications occur in periodontal tissues during the periodontitis process. Currently, DNA methylation is the most studied epigenetic modification associated with periodontitis (16). DNA methylation is a widespread epigenetic alteration in eukaryotic cells, involving the attachment of methyl groups to cytosine residues within CpG dinucleotides. This modification can be either hypermethylation or hypomethylation, leading to the repression or activation of certain genes (17). DNA methylation of cytokine-encoding genes has been found in periodontal tissues of patients with periodontitis (18). For instance, the IL6 gene expression in the gingival tissues of patients with periodontitis was elevated compared to healthy controls (19). In addition, DNA methylation affects genes encoding interferons and chemokines (20). Recently, researchers investigated CpG methylation of 22 inflammatory candidate genes (21). These findings may provide some new insights into the relationship between altered methylation of encoded genes and periodontitis.

**Figure 1:**
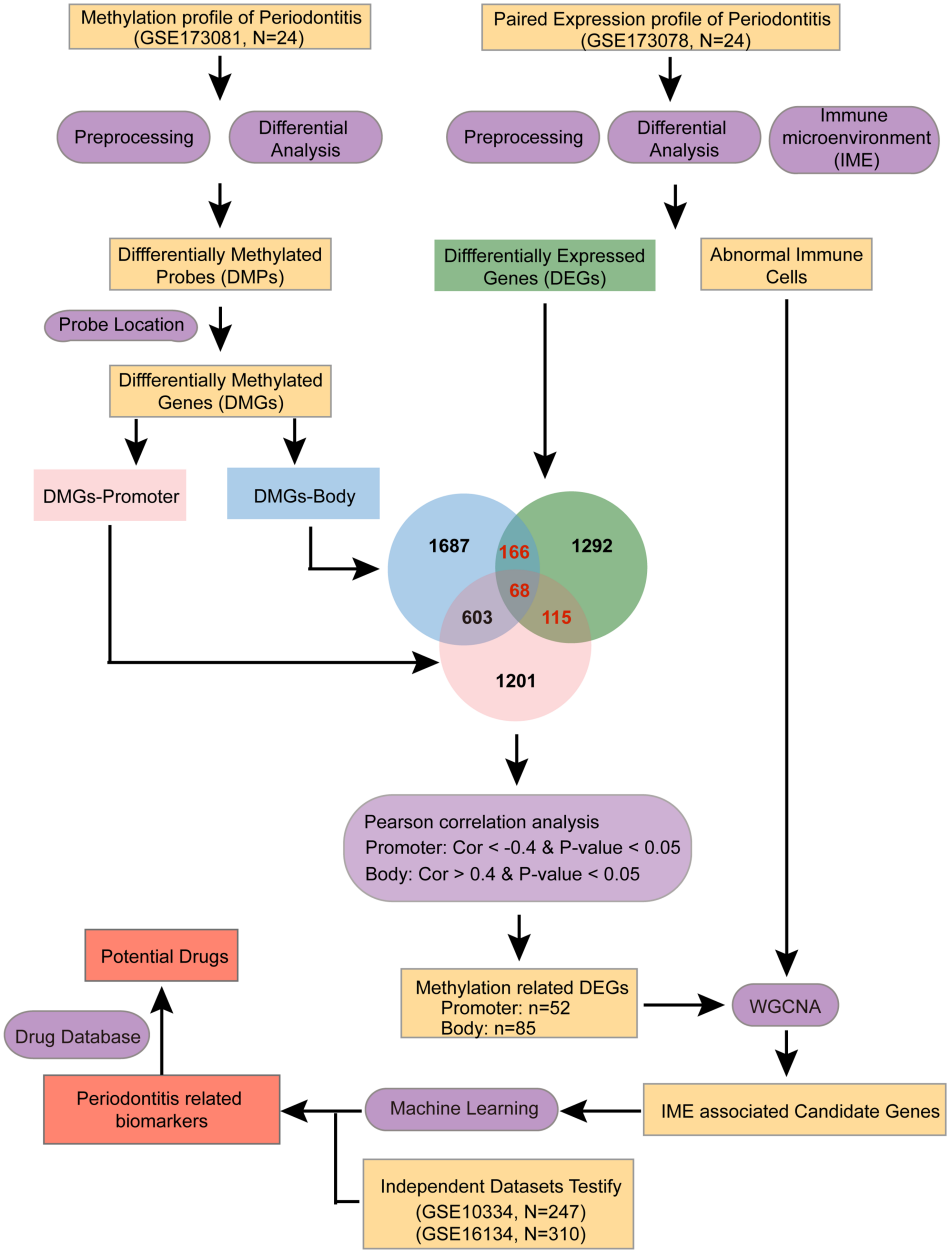
Workflow of the study.

In this study, we hypothesize that integrating transcriptomic and DNA methylation profiles will reveal novel immune-related biomarkers and mechanistic links in periodontitis. To confirm it, we systematically integrated periodontitis-associated transcriptome and DNA methylation data to explore the immune microenvironment of periodontitis. We aimed to identify key immune biomarkers in multiple omics dimensions using a range of bioinformatics approaches (Figure 1). These findings may offer new insights for the development of diagnostic and therapeutic biomarkers for periodontitis.

## Methods

### Data source

DNA methylation and corresponding mRNA expression data from periodontitis patients were retrieved from the GEO database under accession numbers GSE173081 (DNA methylation, N_total_ = 24, N_periodontitis_ = 12, and N_healthy_ = 12) and GSE173078 (mRNA expression, N_total_ = 24, N_periodontitis_ = 12, and N_healthy_ = 12). Two additional independent datasets, GSE16134 (testing dataset1, N_total_ = 310, N_periodontitis_ = 241, and N_healthy_ = 69) and GSE10334 (testing dataset2, N_total_ = 247, N_periodontitis_ = 183, and N_healthy_ = 64), were used for testing. All FPKM expression values were normalized using a log2 transformation. All these datasets are publicly available and unrestricted re-use is permitted via the open license of GEO database.

### DNA methylation profiles

The Illumina Human Methylation EPIC Array was used to analyze the methylation status of periodontitis patients (N_total_ = 24, N_periodontitis_ = 12, and N_healthy_ = 12). This bead chip covers more than 810,000 methylation sites per sample. The raw data were processed by the following steps: firstly, probes with a null value and located in sex chromosomes were removed. Then, probes that mapped to multiple genes or were not mapped to genes or containing SNPs were removed.

The *minfi* R package was used for the normalization of the raw Methylation EPIC Array data. Probes with a *p*-value < 0.05 and absolute detabeta (|Δβ|) > 0.1 were considered differentially methylated.

### Immune microenvironment analysis

The *xcell* R package was employed to estimate the abundance of 64 immune cell types in periodontitis patients, including various T-cell subtypes and other immune cells such as B cells, NK cells, monocytes, and macrophages. The abundance of immune cells in periodontitis patients was compared to that in healthy individuals to identify distinctive features for further investigation.

### Differential expression analysis

The *limma* R package was employed to analyze gene expression differences between periodontitis and control groups. Differentially expressed mRNAs were identified with an adjusted *p*-value < 0.05 and an absolute log2 fold change ≥ 0.263 (22). Subsequently, Pearson correlation analysis was conducted to assess the relationship between DNA methylation levels and gene expression. Only correlations with an absolute Pearson coefficient above 0.4 and a *p*-value below 0.05 were considered significant.

### WGCNA

Co-expression networks were constructed using the WGCNA R package to analyze candidate genes showing correlated patterns in both methylation and expression levels, alongside abnormal immune cell types in periodontitis patients. In this study, hierarchical clustering was used to group genes with similar expression patterns. These gene clusters were then linked to the altered immune cells in patients, and the most relevant genes within these clusters were selected for further investigation.

### Machine learning

The *randomForest* R package was employed to build a periodontitis prediction model using the random forest method, which involved training and testing categorical models to identify gene combinations with high discriminatory power for distinguishing periodontitis from normal groups. The key genes were identified using an SVM algorithm with the *e1071* R package to construct an optimal diagnostic model.

### Function enrichment analysis

We extracted all differentially expressed genes (DEGs) and differentially methylated genes (DMGs) for further functional enrichment analysis using the Metascape webserver. Enrichment analysis was conducted for KEGG pathways and Hallmark gene sets, with functions selected based on a false discovery rate of less than 0.05.

Statistical analyses were performed using R software (version 4.3.2). A *t*-test was used to assess differences between the two groups, and a *p*-value of less than 0.05 was considered statistically significant.

## Results

### Differently expressed and differentially methylated genes are associated with inflammatory and immune-related pathways in periodontitis

**Figure 2:**
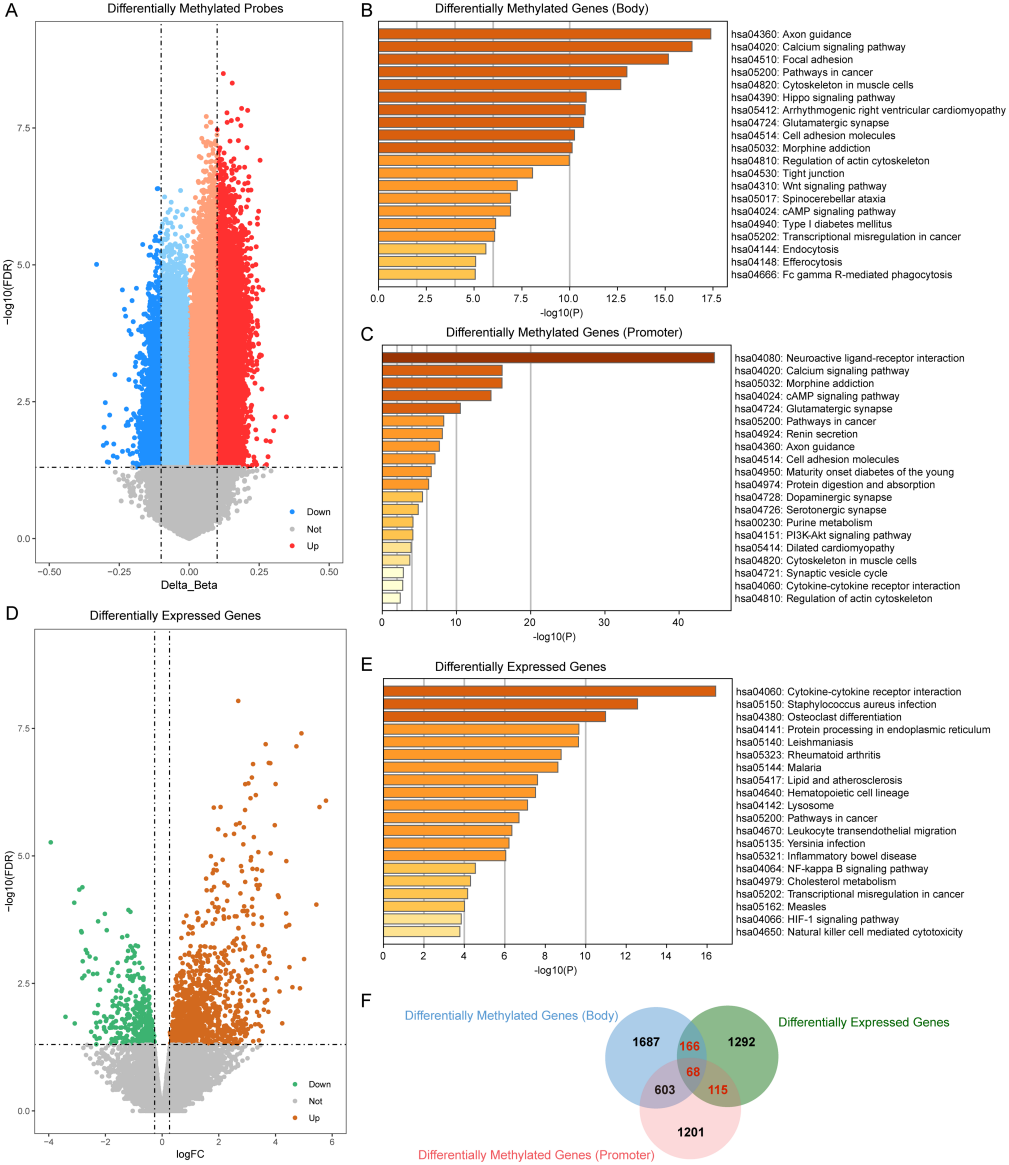
Transcriptome- and DNA mathylation-based screening of periodontitis. **(A)** Volcano plot of differentially methylated probes (periodontitis vs. control). **(B)** Enrichment analysis of differentially methylated probes which located at gene body region. **(C)** Enrichment analysis of differentially methylated probes which located at gene promoter region. **(D)** Volcano plot of differentially expressed genes (periodontitis vs. control). **(E)** Enrichment analysis of differentially expressed genes. **(F)** Venn plot showed the overlap of differentially methylated probes and differentially expressed genes.

We first assessed methylation levels in patients with periodontitis. First, we performed differential analysis of the EPIC methylation array and obtained a total of 23,528 differentially methylated sites (*p* < 0.05, |Δβ| > 0.1). Subsequently, we categorized the differentially methylated probes into promoter region probes (TSS200, TSS1500, 1stExon) and body region probes based on their location in the genome. Among them, there are 5,152 differentially methylated promoter region probes distributed on 2,489 genes and 4,814 differentially methylated body region probes, which fell on 2,784 genes (Figure 2A). Subsequently, we performed enrichment analysis of these differentially methylated genes. The results showed that the differentially methylated genes in the body region were mainly enriched in the Calcium signaling pathway, Wnt signaling pathway and other inflammation-related pathways (Figure 2B), while the differentially methylated genes in the promoter region were mainly enriched in the cMAP signaling pathway, the PI3K-Akt signaling pathway, and the Cytokine-cytokine receptor interaction. Receptor interaction and other immune-related pathways (Figure 2C).

To further elucidate the functional impact of these epigenetic modifications, we next examined the gene expression profiles in periodontitis patients. We analyzed the gene expression data of periodontitis patients to screen for genes abnormally expressed in periodontitis (|log2FC| > 0.263, *p* < 0.05). We screened a total of 1,641 differential expressed genes, of which 398 were abnormally down-regulated and 1,243 were abnormally up-regulated (Figure 2D). Enrichment analysis of these differential genes showed that periodontitis-associated aberrantly expressed genes were mainly enriched in pathways such as Cytokine-cytokine receptor interaction, NF-kappa B signaling pathway and HIF-1 signaling pathway (Figure 2E). The NF-kappa B signaling pathway, in particular, plays a pivotal role in orchestrating inflammatory responses. Activation of NF-kappa B leads to the transcription of a variety of cytokines and chemokines that mediate inflammation, which is critical in the progression of periodontitis. This pathway can contribute to the persistence of inflammation, thereby exacerbating tissue destruction and bone resorption observed in periodontitis.

In our integrated analysis, we identified 349 genes that were both differentially methylated and differentially expressed. Notably, several key genes involved in inflammation and immune regulation were among these 349 genes. For example, MMP9, a matrix metalloproteinase known for its role in tissue remodeling and inflammatory processes, has been implicated in periodontal tissue degradation. Similarly, CD86, a critical co-stimulatory molecule involved in T-cell activation, and PTPRC (CD45), a regulator of immune cell signaling, underscore the immune involvement in periodontitis. Other genes such as IL2RA and IL21R are central to immune cell differentiation and activation, while FAM43B and FOXA3 have emerged as potential diagnostic markers in our analysis. These gene-specific findings reinforce the biological relevance of our integrated analysis and suggest that the dysregulation of these key genes may contribute significantly to the pathogenesis of periodontitis (Figure 2F).

### Altered immune cells in periodontitis linked to differentially expressed gene modules regulated by aberrant methylation

**Figure 3:**
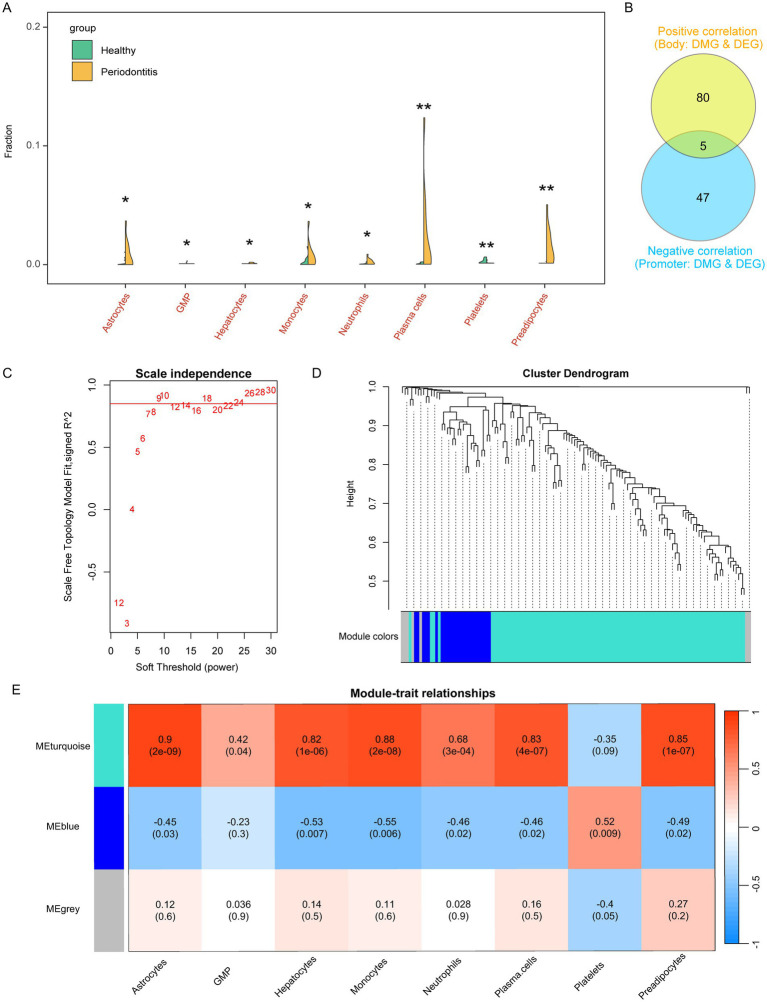
Assessment of the immune microenvironment for periodontitis and screening of relevant gene modules. **(A)** Violin plot showed the altered immune cells of periodontitis. GMP, Granulocyte-Macrophage Progenitor. **(B)** Correlation ship between differentially methylated probes and differentially expressed genes. **(C)** Power of WGCNA co-expression network. **(D)** Cluster dendrogram of candidate genes which were correlated in DNA methylation and gene expression in periodontitis. **(E)** Immune cells associated co-expression modules in periodontitis.

We evaluated the immune microenvironment of periodontitis patients based on the xcell algorithm. The results showed that the abundance of immune cells such as Astrocytes, Granulocyte-Macrophage Progenitor (GMP), Hepatocyte, Monocyte, Neutrophil, Plasma cells, and Prevacidocytes was significantly increased while Platelets were significantly decreased in periodontitis patients (Figure 3A; Supplementary Figure 1). It is important to note that the detection of hepatocyte signatures in gingival tissue is unexpected. This may be due to the inherent limitations of the xCell algorithm, which relies on gene expression profiles that can sometimes overlap among different cell types. The “Hepatocyte” signal observed might represent a similar cell population with a related expression profile rather than true hepatocytes. Further experimental validation is needed to clarify this observation. It should be noted that the detection of “hepatocyte” and “platelet” signatures likely reflects algorithmic limitations of bulk transcriptomic deconvolution rather than the true presence of these cell types in gingival tissue.

These alterations in immune cell composition suggest an imbalance in immune regulation, potentially driven by underlying epigenetic changes. Subsequently, we further screened the aberrant genes regulated by methylation, and the screening criteria were, body region differentially methylated genes, whose methylation level was positively correlated with the expression level (R > 0.4, *p* < 0.05), and promoter region differentially methylated genes, whose methylation level was negatively correlated with the expression level (R < −0.4, *p* < 0.05). These correlation thresholds were chosen to ensure a moderate to strong association between methylation changes and gene expression regulation while minimizing false positives. Previous studies have used similar cutoffs to establish meaningful methylation-expression relationships in disease contexts (23, 24). Finally, we screened 132 eligible candidate genes (Figure 3B).

To connect these findings with the observed immune cell alterations, we investigated whether the aberrantly methylated genes might drive changes in immune cell profiles. We performed WGCNA analysis to screen the co-expression modules of altered immune cells based on the expression levels of these 132 candidate genes with the abundance of the above mentioned 8 altered immune cells associated with periodontitis. The results showed that there were three expression patterns of these candidate genes (Figures 3C,D), among which, the MEturquoise module (contains 100 candidate genes) was significantly associated with abnormally elevated immune cells such as Astrocytes, GMP, Hepatocyte, Monocyte, Neutrophil, Plasma cells, and Previpocytes. correlated, while MEblue (contains 25 candidate genes) significantly correlated with Astrocytes, Hepatocyte, Monocyte, Neutrophil, Plasma cells, Previpocytes and Platelets (Figure 3E; Supplementary Table S1). These findings indicate that epigenetic regulation, as reflected by aberrant methylation, may influence immune cell composition by modulating the expression of gene modules relevant to immune functions.

### Machine learning-based screening for multi-omics diagnostic biomarkers in periodontitis patients

**Figure 4:**
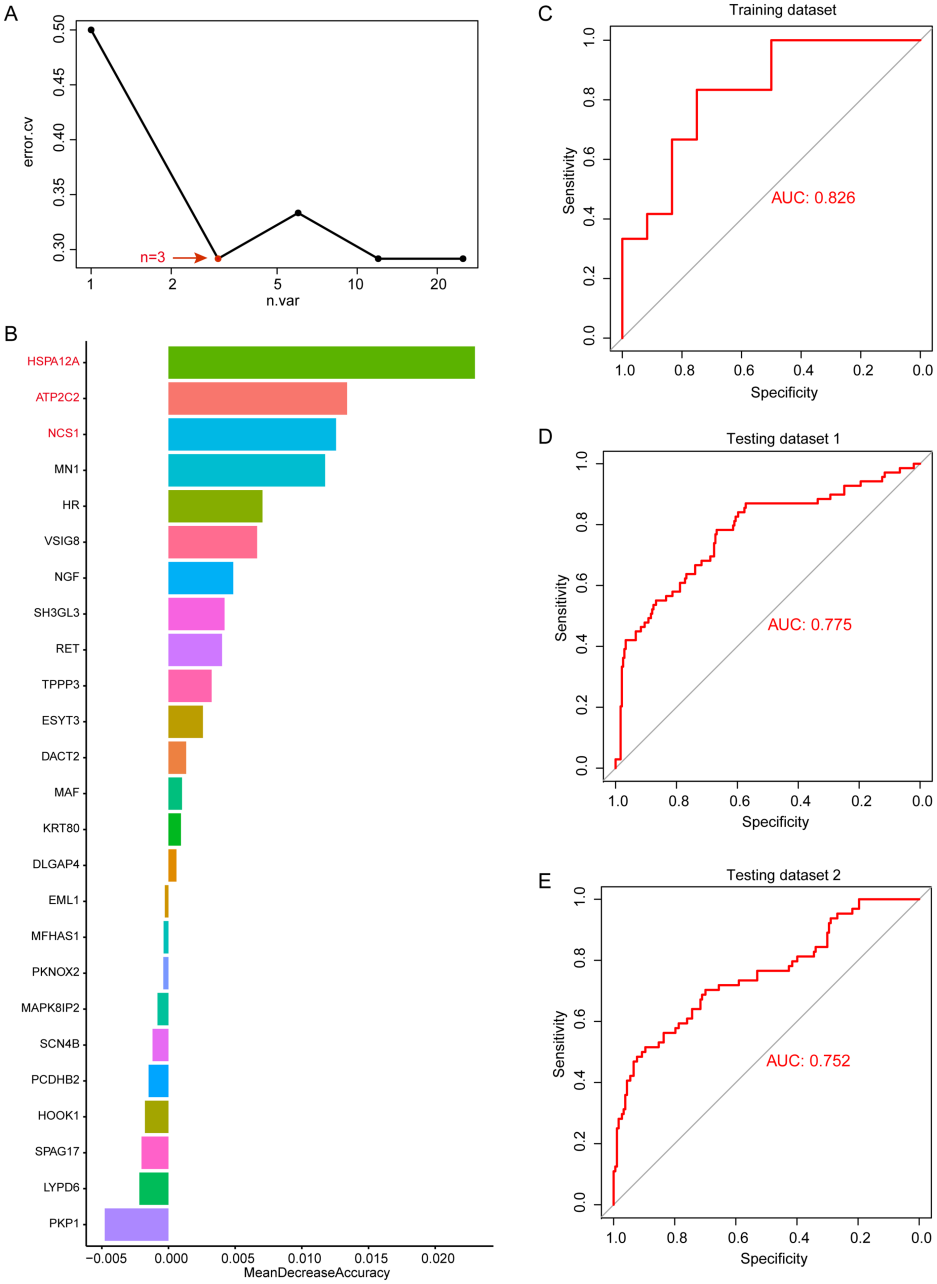
Random forest analysis in MEblue. **(A)** error.cv. plot of random forest analysis. Here, the random forest model has the optimal classification efficacy when the number of genes in the model reaches 3. **(B)** Mean decrease accuracy of random forest model. **(C)** SVM performance in training datasets. **(D)** SVM performance in testing datasets 1. **(E)** SVM performance in testing datasets 2.

**Figure 5:**
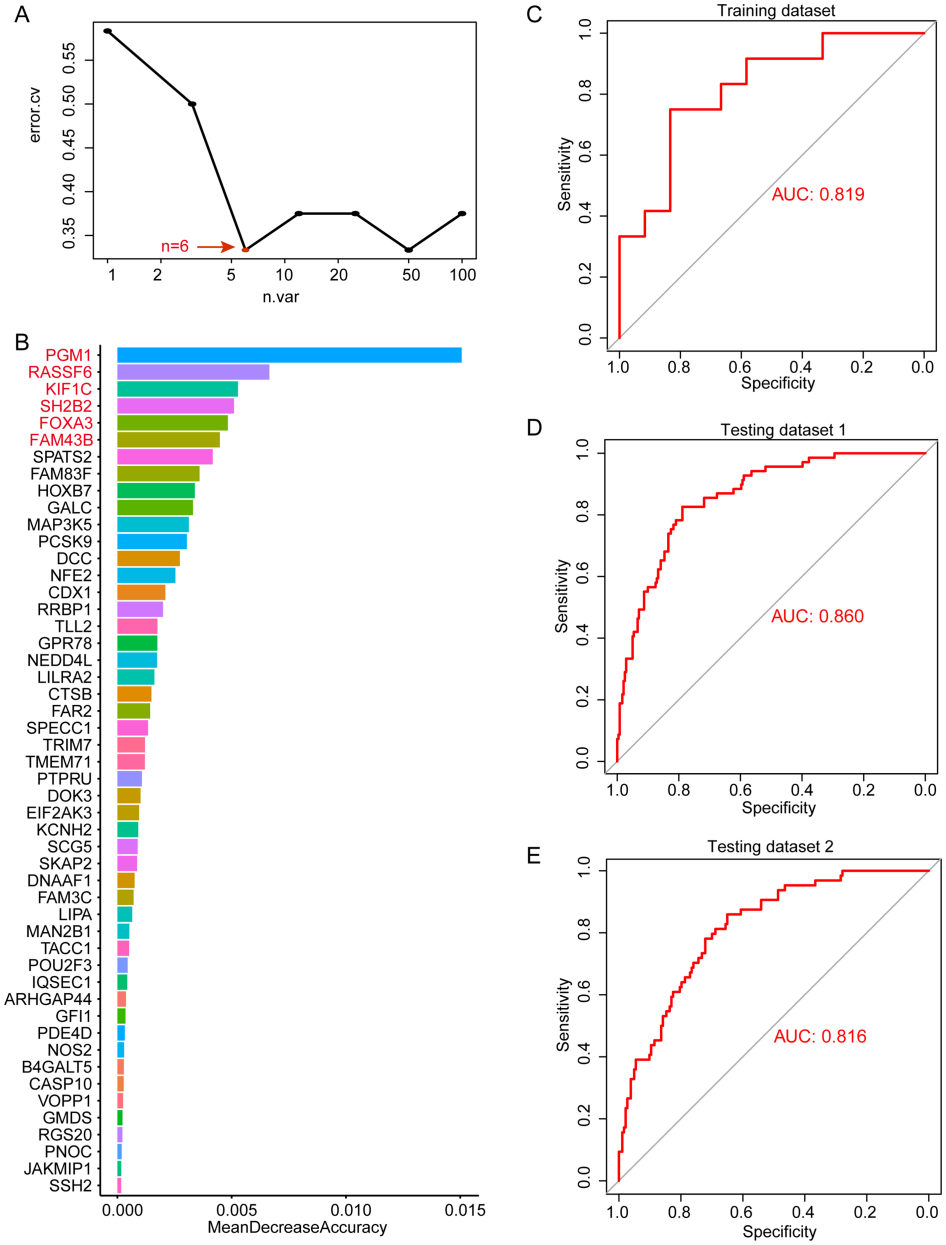
Random forest analysis in MEturquoise. **(A)** error.cv. plot of random forest analysis. Here, the random forest model has the optimal classification efficacy when the number of genes in the model reaches 6. **(B)** Mean decrease accuracy of random forest model. **(C)** SVM performance in training datasets. **(D)** SVM performance in testing datasets 1. **(E)** SVM performance in testing datasets 2.

Through WGCNA analysis, we found that MEblue and MEturquoise are associated with altered immune cells in periodontitis patients. Among them, MEblue contains 25 candidate genes while MEturquoise contains 100 candidate genes. These gene modules exhibited significant correlations with immune cell types that are dysregulated in periodontitis, including monocytes, neutrophils, and plasma cells (Figure 3E). The enrichment analysis of these genes revealed their involvement in immune-related pathways (such as leukocyte activation, toll-like receptor 2 signaling pathway), further underscoring their biological relevance (Supplementary Figure 2). The strong correlation between these genes and immune cell alterations suggests their potential role in immune dysregulation and inflammation in periodontitis. Thus, we selected these genes for machine learning analysis to identify the most informative biomarkers for disease classification. We first performed random forest modeling for the 25 genes in the MEblue module. The results show that the random forest model has the optimal classification efficacy when the number of genes in the model reaches 3 (Figure 4A). Subsequently, we show the gene scores for each node in the random forest and select the top3 genes (HSPA12A, ATP2C2, and NCS1) (Figure 4B). These three genes have been previously implicated in periodontitis-related processes. For instance, HSPA12A is known to regulate inflammatory responses (25), ATP2C2 plays a critical role in immune microenvironment (26), and NCS1 is associated with immunotherapy and prognosis of cancer (27). Next, we construct a classification model based on SVM for these top3 genes. The results show that in the training set, the classification efficiency of this 3-gene model reaches 0.826 (AUC = 0.826, Figure 4C), while in the testing dataset1, the AUC of this model is also as high as 0.775 (Figure 4D), and in the testing dataset2, the AUC value is 0.752 (Figure 4E). This suggests that this 3-gene model has a better diagnostic efficacy for periodontitis patients. In the MEturquoise module, we found that the 6-gene random forest model had the best classification efficacy (Figure 5A). We then ranked the genes in the model based on importance and selected the top6 genes (PGM1, RASSF6, KIF1C, SH2B2, FOXA3, and FAM43B) (Figure 5B). The six genes selected from the MEturquoise module are also intricately linked to immune and inflammatory pathways. For example, PGM1 and RASSF6 are associated with macrophage (28). KIF1C could regulate the podosome dynamics in macrophages (29). The immunologic significance of SH2B2 is related to the invasion of colon adenocarcinoma (30). FOXA3 is a transcriptional activator that is associated with signal transduction in tumors (31). Additionally, FAM43B could repress the cell proliferation and is regulated by DNA methylation (32). Similarly, we construct SVM classifiers based on these 6 candidate genes. The results show that this model has AUC = 0.819 (Figure 5C) in the training set, while in testing dataset1 and testing dataset2, the AUC is 0.860 (Figure 5D) and 0.816 (Figure 5E), respectively. Detailed performance metrics are also summarized in Supplementary Table S2. These results suggesting that these models have effective efficacy for periodontitis diagnosis.

### Identification of target drugs for periodontitis patients based multi-omics diagnostic biomarkers

**Figure 6:**
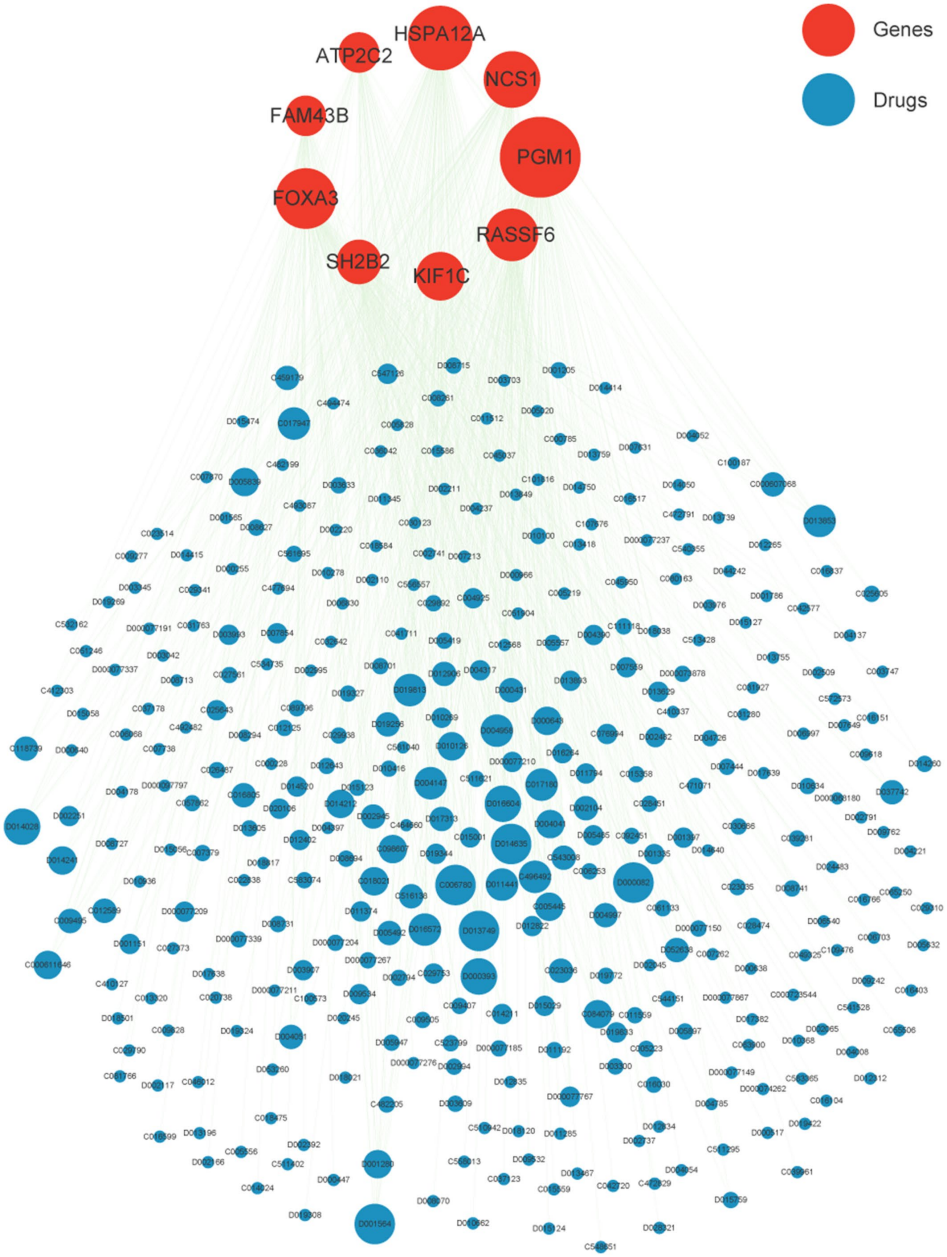
Drug network of nine crucial genes. Red nodes represent nine crucial gene while blue is potential target drugs. The node size indicates the degree of the network.

Based on epigenome and transcriptomics, we screened 9 periodontitis diagnostic genes in the periodontitis immune microenvironment. Subsequently, we further explored potential target drugs for these 9 genes. We constructed a drug-targeting network for these genes based on the CTD database and identified 345 drugs/compounds targeting these 9 genes (Figure 6). Through further network analysis, we screened out 3 drugs/compounds targeting all 9 genes simultaneously: bisphenol A, Acetaminophen and Valproic Acid (Supplementary Table S3). Bisphenol A, though primarily considered an environmental contaminant, has been implicated in immune modulation and inflammatory responses (33). Acetaminophen is widely used as an analgesic and has been shown to modulate oxidative stress pathways, which are relevant in periodontitis pathology (34). Valproic acid, a histone deacetylase inhibitor, has demonstrated anti-inflammatory effects and potential benefits in immune-related conditions (35). These insights support the relevance of these drugs in the context of periodontitis and highlight their possible regulatory roles in disease-associated pathways. Among them, Acetaminophen and Valproic Acid are FDA-approved drugs with better results in analgesia. In this study, we found for the first time that they are associated with periodontitis-related targets, which provides a new idea for the subsequent screening of potential periodontitis-related drugs. Their repurposing for periodontitis could offer advantages such as well-characterized pharmacokinetics and widespread clinical availability. However, the potential off-target effects and adverse reactions-such as hepatotoxicity for Acetaminophen and the broad systemic effects associated with Valproic Acid-necessitate further investigation in the context of periodontitis. Additional preclinical studies and clinical trials are warranted to optimize dosing, evaluate long-term safety, and establish their efficacy as adjuncts in periodontitis management.

## Discussion

While numerous studies have highlighted the immune microenvironment’s involvement in the development of periodontitis, the exact mechanisms through which it affects the onset and progression of the disease are still not fully understood (21–23). It has been found that periodontitis is not only affected by the transcriptional level but also involves epigenetic alterations. Nevertheless, there are limited studies that have identified immune-related genes linked to periodontitis across various histological layers, which could potentially serve as important clinical biomarkers for the disease. In this study, we first investigated the immune microenvironment of periodontitis and identified candidate genes that showed both abnormal methylation and expression patterns in periodontitis samples, utilizing epigenomic and transcriptomic approaches. Subsequently, through a multi-dataset machine learning algorithm, we further narrowed down these candidate genes to nine key genes with diagnostic efficacy for periodontitis, namely, ATP2C2, FAM43B, FOXA3, HSPA12A, KIF1C, NCS1, PGM1, RASSF6, and SH2B2. We then further analyzed these genes by network pharmacology to screen for their potential drug targets. This study revealed the association of key genes related to the immune microenvironment with periodontitis at the epigenetic and transcriptional levels, and screened for drug targets that could regulate these key genes through the drug target network. Our research offers significant insights into the potential use of these key genes as diagnostic and therapeutic markers for improving the clinical management of periodontitis.

ATP2C2 is involved in calcium transmembrane transport, intracellular calcium ion homeostasis, and manganese ion transport (36). FAM43B has been found to control innate immunity through Epigenetic Regulation (37). FOXA3 encodes a forkhead-like DNA-binding protein that interacts with chromatin. It also plays a role in the regulation of metabolism as well as organ differentiation. FOXA3 methylation has been found to cause dedifferentiation and sorafenib resistance in hepatocellular carcinoma (38). HSPA12A, which is predicted to have ATP-binding activity and is located in extracellular exosomes, was found to promote nuclear PKM2-mediated polarization of M1 macrophages (39). The protein encoded by KIF1C belongs to a family of kinesin-like proteins that transport APC-dependent mRNAs to cellular protrusions (40) and can re-localize GLUT4 to immune-modification-positive cell sites (41). NCS1, a member of the neuronal calcium sensor gene family, is a key Ca2 + −binding protein thought to play a role in cell proliferation and immune infiltration (27). The protein encoded by this gene is an isoform of phosphoglucomutase (PGM) and is associated with M2 macrophages and TFH cells and their surface markers CD163 and CXCR5 (42). RASSF6 encodes a member of the Ras-associated structural domain family (RASSF), and the protein encoded by this gene is a Ras effector protein that induces apoptosis. In acute lymphoblastic leukemia (ALL), there is a high prevalence of aberrant RASSF6 promoter methylation, and its DNA methylation status has the potential to serve as a biomarker for assessing MRD levels in ALL patients (43). SH2B2 encodes a protein expressed in B lymphocytes that undergoes tyrosine phosphorylation in response to B cell receptor stimulation and plays a role in signaling in the Shc/Grb2 pathway (44).

In conclusion, through our integration of DNA methylation profiles and transcriptomes of periodontitis patients, we assessed the immune microenvironment of periodontitis patients and screened nine diagnostic markers related to periodontitis patients based on machine-learning algorithms, and screened for relevant targeted drugs. This finding will provide new insights for subsequent diagnosis and treatment of periodontitis. Furthermore, our study builds on previous research using machine learning to identify biomarkers in immune-related diseases. For instance, studies on interactomic hub gene prediction in PBMCs for type 2 diabetes mellitus, dyslipidemia, and periodontitis have demonstrated the potential of network-based approaches in identifying key regulatory genes (45). Additionally, machine learning models for predicting rheumatoid arthritis based on ACPA autoantibody development in the presence of non-HLA gene polymorphisms highlight the utility of such methods in complex diseases (46). Similarly, the prediction of interactomic hub genes in rheumatoid arthritis using peripheral mononuclear cells underscores the importance of transcriptomic and network-based analyses in understanding immune-related pathologies (47). Our study contributes to this growing body of research by identifying key diagnostic genes and their potential drug interactions in periodontitis. Meanwhile, our diagnostic models, with AUC values ranging from 0.75 to 0.86, compare favorably with existing periodontitis biomarkers, which often rely on single-parameter assessments such as probing depth, clinical attachment loss, or inflammatory mediators in gingival crevicular fluid (48). The integration of epigenetic and transcriptomic data in our models not only improves diagnostic accuracy but also captures the complexity of the disease’s molecular basis. This multi-omics approach allows for a more comprehensive evaluation of the disease state and may facilitate the development of personalized treatment strategies. The identification of these drugs through a multi-omics approach presents a novel strategy for periodontitis therapy. In terms of efficacy, the FDA-approved drugs Acetaminophen and Valproic Acid have well-documented pharmacological profiles that may enhance their potential as adjunct therapies. They offer the possibility of modulating key molecular mechanisms underlying periodontitis, such as oxidative stress and immune regulation. However, while conventional therapies focus on bacterial control and symptomatic relief, these drugs may provide benefits by directly impacting the disease’s molecular drivers. Regarding safety, current standard therapies generally have minimal systemic side effects but may not fully address the inflammatory and tissue-degradative components of periodontitis. In contrast, the off-target effects of Acetaminophen (e.g., hepatotoxicity) and Valproic Acid (e.g., gastrointestinal and metabolic disturbances) require careful dosing and monitoring.

Overall, these findings underscore the clinical and biological significance of integrating multi-omics data to identify potential therapeutic agents. The approach not only enhances our understanding of periodontitis pathogenesis but also opens new avenues for developing targeted interventions that may complement existing treatment modalities.

Despite the promising findings of our study, several limitations should be acknowledged. First, our analyses relied on publicly available datasets with relatively small sample sizes, which may limit the generalizability of the results. The lack of detailed demographic information, such as age and gender, may also introduce selection bias and restrict applicability across broader populations. Future large-scale studies with demographically matched cohorts are warranted to address these concerns. Second, while the use of multi-dataset machine learning improved robustness, potential confounders (including patient demographics, disease severity, and sample processing) could still influence the outcomes. Integrating additional omics layers, such as proteomics and metabolomics, may provide a more comprehensive understanding of periodontitis pathogenesis.

Moreover, the current study provides predictive insights into immune alterations in periodontitis based on bulk transcriptomic deconvolution. However, bulk analyses cannot fully capture the complexity of the immune microenvironment, which ideally requires single-cell transcriptomic and spatially resolved approaches. Importantly, all conclusions are computationally derived without protein-level or *in vivo* validation. Future research should therefore include experimental confirmation, such as immunohistochemistry, flow cytometry, and animal models, to validate the biological and therapeutic relevance of the identified biomarkers and drug candidates. Specifically, preclinical testing of acetaminophen and valproic acid will be crucial to determine their mechanistic roles and feasibility as adjunctive therapies for periodontitis.

## Conclusion

In conclusion, through our integration of DNA methylation profiles and transcriptomes of periodontitis patients, we assessed the immune microenvironment of periodontitis patients and screened nine diagnostic markers related to periodontitis patients based on machine-learning algorithms, and screened for relevant targeted drugs. This finding will provide new insights for subsequent diagnosis and treatment of periodontitis.

## Data Availability

The datasets used and analyzed during the current study are downloaded from GEO database under accession numbers: GSE173081, GSE173078, GSE10334, and GSE16134.

## Supplementary material

SUPPLEMENTARY FIGURE 1 Assessment of the immune microenvironment for periodontitis. Violin plot showed the abundance of immune cells in periodontitis. DC, Dendritic Cell; CLP, Common Lymphoid Progenitor; CMP, Common Myeloid Progenitor; GMP, Granulocyte-Macrophage Progenitor; HSC, Hematopoietic Stem Cell; MEP, Megakaryocyte-Erythroid Progenitor; MPP, Multipotent Progenitor; MSC, Mesenchymal Stem Cell; NKT, Natural Killer T Cell.

SUPPLEMENTARY FIGURE 2 Enrichment analysis of genes in MEturquoise and MEblue. **(A)** Enrichment analysis of 100 module genes in MEturquoise. **(B)** Enrichment analysis of 25 module genes in MEblue.

## Glossary

WGCNA: Weighted correlation network analysis
SVM: Support Vector Machine
AUC: Area Under the Curve
PGE_2_: prostaglandin E_2_
IL-1β: interleukin-1β
TNF-*α*: tumor necrosis factor-α; IL-8
IFN-*γ*: interferon-γ
FPKM: Fragments Per Kilobase per Million
DEGs: differentially expressed genes
DMGs: differentially methylated genes
FC: Fold-Change value
DC: Dendritic Cell
CLP: Common Lymphoid Progenitor
CMP: Common Myeloid Progenitor
GMP: Granulocyte-Macrophage Progenitor
HSC: Hematopoietic Stem Cell
MEP: Megakaryocyte-Erythroid Progenitor
MPP: Multipotent Progenitor
MSC: Mesenchymal Stem Cell
NKT: Natural Killer T Cell
CTD: Comparative Toxicogenomics Database
PGM: phosphoglucomutase
RF: random forest
RASSF: Ras-associated structural domain family
GEO: Gene Expression Omnibus database
