# Supplementary figures and images for: Multi-omics integration to identify immune-associated biomarkers and potential therapeutics in periodontitis
Source: Front Med (Lausanne). 2025 Oct 10;12:1640961. doi: 10.3389/fmed.2025.1640961 (PMC12549644; doi:10.3389/fmed.2025.1640961)

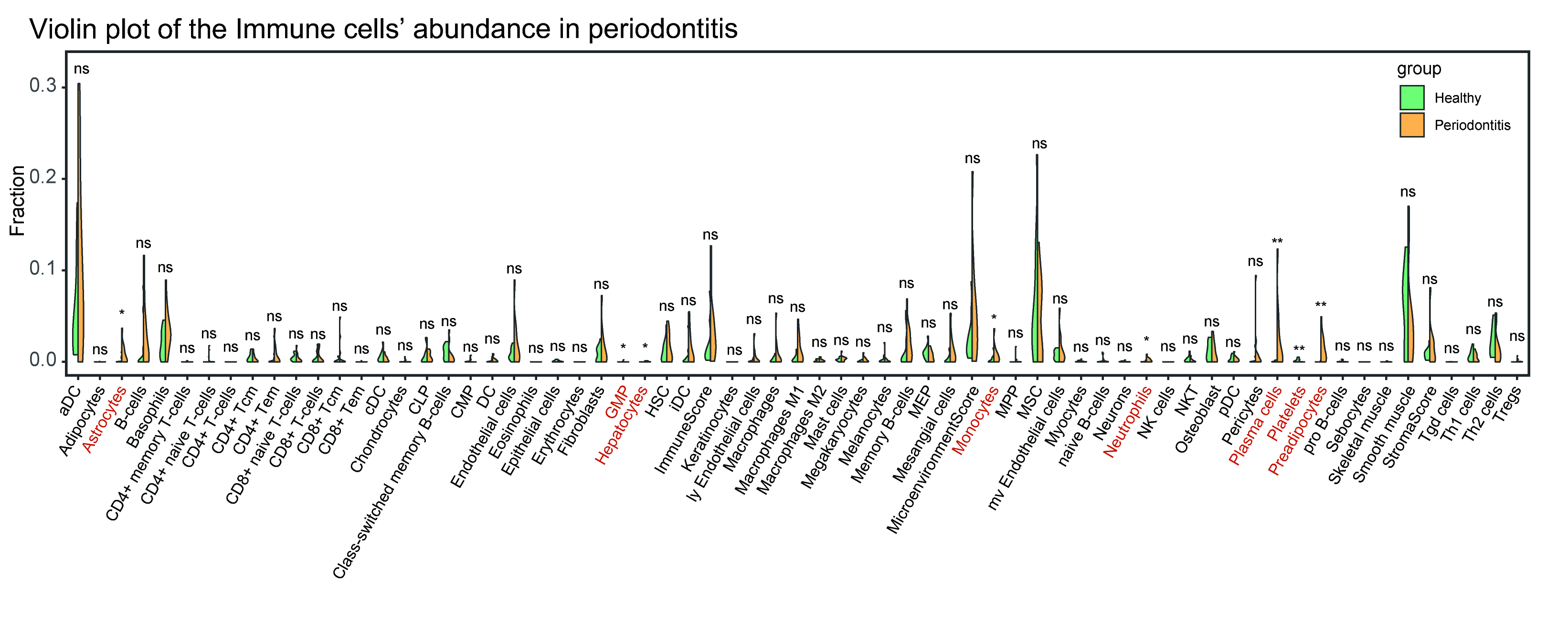

Supplement: SUPPLEMENTARY FIGURE 1 — Assessment of the immune microenvironment for periodontitis. Violin plot showed the abundance of immune cells in periodontitis. DC, Dendritic Cell; CLP, Common Lymphoid Progenitor; CMP, Common Myeloid Progenitor; GMP, Granulocyte-Macrophage Progenitor; HSC, Hematopoietic Stem Cell; MEP, Megakaryocyte-Erythroid Progenitor; MPP, Multipotent Progenitor; MSC, Mesenchymal Stem Cell; NKT, Natural Killer T Cell. [file Image_1.tif]

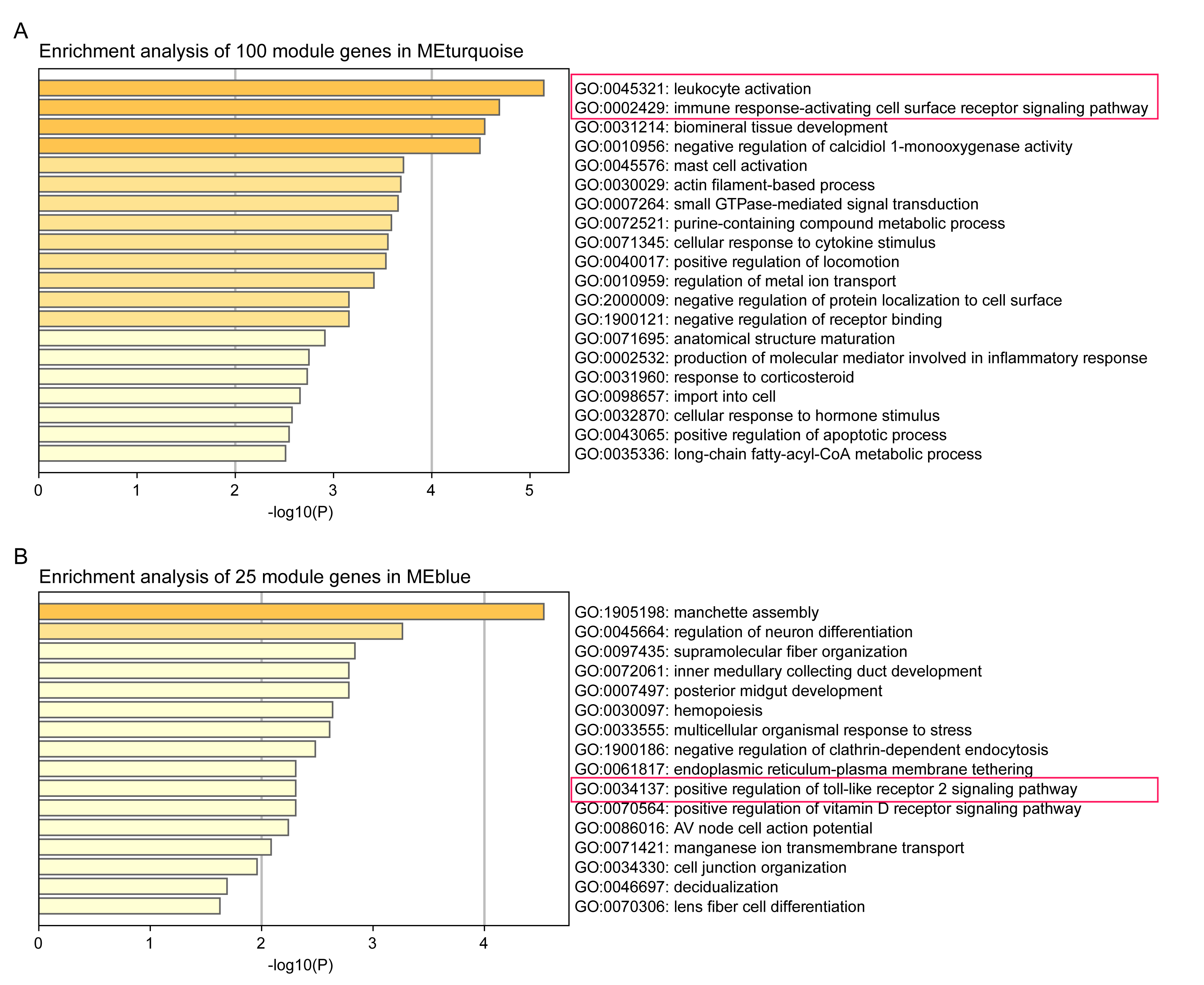

Supplement: SUPPLEMENTARY FIGURE 2 — Enrichment analysis of genes in MEturquoise and MEblue. (A) Enrichment analysis of 100 module genes in MEturquoise. (B) Enrichment analysis of 25 module genes in MEblue. [file Image_2.tif]
